# Program Code Generator for Cardiac Electrophysiology Simulation with Automatic PDE Boundary Condition Handling

**DOI:** 10.1371/journal.pone.0136821

**Published:** 2015-09-10

**Authors:** Florencio Rusty Punzalan, Yoshitoshi Kunieda, Akira Amano

**Affiliations:** 1 Department of Bioinformatics, College of Life Sciences, Ritsumeikan University, Shiga, Japan; 2 Department of Computer Science, College of Information Science and Engineering, Ritsumeikan University, Shiga, Japan; University of Oxford, UNITED KINGDOM

## Abstract

Clinical and experimental studies involving human hearts can have certain limitations. Methods such as computer simulations can be an important alternative or supplemental tool. Physiological simulation at the tissue or organ level typically involves the handling of partial differential equations (PDEs). Boundary conditions and distributed parameters, such as those used in pharmacokinetics simulation, add to the complexity of the PDE solution. These factors can tailor PDE solutions and their corresponding program code to specific problems. Boundary condition and parameter changes in the customized code are usually prone to errors and time-consuming. We propose a general approach for handling PDEs and boundary conditions in computational models using a replacement scheme for discretization. This study is an extension of a program generator that we introduced in a previous publication. The program generator can generate code for multi-cell simulations of cardiac electrophysiology. Improvements to the system allow it to handle simultaneous equations in the biological function model as well as implicit PDE numerical schemes. The replacement scheme involves substituting all partial differential terms with numerical solution equations. Once the model and boundary equations are discretized with the numerical solution scheme, instances of the equations are generated to undergo dependency analysis. The result of the dependency analysis is then used to generate the program code. The resulting program code are in Java or C programming language. To validate the automatic handling of boundary conditions in the program code generator, we generated simulation code using the FHN, Luo-Rudy 1, and Hund-Rudy cell models and run cell-to-cell coupling and action potential propagation simulations. One of the simulations is based on a published experiment and simulation results are compared with the experimental data. We conclude that the proposed program code generator can be used to generate code for physiological simulations and provides a tool for studying cardiac electrophysiology.

## Introduction

Over the past few decades, experiments in cardiac electrophysiology have been increasingly supplemented by computational models, ranging from a single cell to tissue and whole-heart simulation. These mathematical models of cardiac electrophysiology usually consist of partial differential equations (PDEs) coupled with a system of ordinary differential equations (ODEs) that describe the biological function model and supplemented by boundary conditions.

Lumped parameter systems described with ODEs have been widely used in biological function models due to their ease of modelling and high analyticity [1]. However, tissue to whole-heart simulation requires a distributed parameter system described with PDEs. This system can describe the distribution of physiological structures and spatial localization of intracellular materials. This includes the handling of boundary conditions and, in cases like pharmacokinetics simulation, distributed parameters that vary through time.

Some examples of lumped parameter system descriptions used to describe biological function models include open standards such as PHML [2], CellML [3] and SBML [4]. FieldML [5] and FML [6], on the other hand, are description languages capable of describing a distributed parameter system. However, these languages are not versatile enough for hybrid lumped-distributed parameter physiological systems. Although FieldML is expected to handle this limitation, this capability is not yet included as of this writing. Widely used tools like OpenCMISS [7] and Chaste [8] support CellML to create multi-cell simulations but users need to have at least some programming background. While Chaste allows multi-scale simulation, it hardcodes the tissue level equations in software.

The structure of simulation programs for lumped parameter systems is relatively homogeneous. However, a distributed parameter system can lead to various solutions depending on the problem’s initial value, boundary condition, spatial discretization, and equation form. The complexity and size of the biological function models in distributed parameter systems can make it difficult for life scientists to implement and create the necessary program code. In addition, changes in the boundary condition can change the calculation order of the simulation equations. These changes often increase the order of complexity and become hard to manage if handled manually.

Future developments in cardiac modelling may require multi-organ simulations and adopt more efficient numerical techniques. To accommodate these and other future advances in simulation, Pitt-Francis et al. [9] and Linge et al. [10] postulated a number of requirements for cardiac simulation software. One of the main requirements is extensibility of software. This can be partially achieved if the code development is flexible with respect to biological function model, geometry, boundary condition and computational model used.

In this study, we propose a code generation system that automatically generates program code for distributed parameter systems described by PDEs and boundary conditions, specifically for cardiac electrophysiology simulation. There have been several automatic program generators for solving PDEs developed over the years. These include OP2 [11], which is an open-source framework for the execution of unstructured grid applications, and Paraiso (PARallel Automated Integration Scheme Organizer) [12], which can generate parallel programs for solving PDEs using explicit schemes. OP2 is a powerful tool for generating parallel implementations of PDE solutions in different back-end hardware platforms—such as a cluster of GPUs. Paraiso takes a problem described in a domain-specific language and generates OpenMP and CUDA programs. However, both of these tools utilize source-to-source translation and compilation, which means the user has to write the application code first (using OP2 API for OP2 and Haskell for Paraiso) before the tools can generate the parallel programs. Our aim is to automate the writing of the application code with the users just supplying a biological function model as input. Our system is restricted to finite difference methods for solving the PDEs.

Through our system, various numerical solution methods can be used to discretize the model equations in a tissue-level simulation. These methods are written in a declarative description style and are not hard-coded into the simulation software. Users can choose one of the provided PDE numerical schemes or they can add one for their simulation. More importantly, since boundary conditions are also described declaratively and handled automatically, different boundary types and combinations can be used without adding to the difficulty of implementation.

This study is an extension of our previous work [13]. The previous work introduced the input files and structure of the code generation system. The implementation of the system described in that study can generate code for simulations with a linear system of equations. This study extends the system capability to support simulations with a nonlinear system of equations. The code generator can now support simultaneous equations in the biological function model as well as implicit PDE numerical schemes. This study also provides details of the boundary condition handling and dependency analysis, which were not described in our previous study.

## Methods

### Tissue-level Electrophysiology Simulation

#### Cardiac modelling overview

In cardiac tissue-to-whole-organ level electrophysiology simulation, equations model the manner in which action potential propagates through the heart. Most of these models represent the tissue either as an anisotropic medium divided into the intracellular and extracellular space or as a functional syncytium where membrane voltage is assumed to propagate smoothly. The former is commonly referred to as the bidomain model [14], while the latter is known as the monodomain model [15]. The bidomain model provides a more detailed model of the cardiac tissue but comes with a much greater computational cost. On the other hand, the monodomain approach offers numerical efficiency and can be sufficient for modelling wave propagation if it is assumed that no applied currents are to be simulated [16]. Either of the two is used in conjunction with biological function models to simulate action potential propagation. We concentrate on the monodomain model as the target of our code generation system.

#### Monodomain model

The monodomain model is a reduction of the bidomain model into a single-space excitable medium, with diffusion and local excitation of membrane voltage. It provides the simplest description of action potential propagation [17]:

$$\begin{array}{c} \frac{\partial V}{\partial t}=\nabla \cdot \left(D\nabla V\right)-\frac{I+J}{C_{m}}, \end{array}$$ (1)

where *V* refers to the membrane voltage, ∇ is the gradient operator, *D* is the diffusion constant, and *C*_*m*_ is the membrane capacitance. The ionic current *I* is a function of the membrane voltage, while *J* is the applied stimulus. The monodomain equation is commonly solved with the Finite Difference Method (FDM) or Finite Element Method (FEM) for the underlying Poisson’s equation.

#### Numerical methods for solving the monodomain equation

The FDM is based on approximating the partial derivatives through difference quotients [18]. It has the advantage of being easy to implement and has been used by many groups for studies of tissue electrophysiology [16, 19]. A disadvantage is that it is difficult to describe complex geometries using FDM. The FEM, on the other hand, is often preferred for solving the monodomain and bidomain equations due to their flexibility in terms of dealing with complex heart geometries. However, FEM requires a much higher computational effort compared to the FDM. For the code generation system, we used FDM to create the program code for solving the PDEs in the monodomain equation. FDM offers a straightforward way of discretizing continuous PDEs and ease of generating program codes automatically.

Finite difference schemes cover a wide array of solutions for PDEs. These solutions can be roughly divided into single-step and multi-step schemes. Single-step or one-level schemes approximate the solution by directly replacing the continuous differential terms with corresponding finite difference terms, with the solution directly calculated from the discretized terms in one time-step [20]. Single-step schemes include the FTCS (forward time centered space) method and Crank-Nicolson method (Table 1). Multi-step schemes, on the other hand, obtain a numerical solution by carrying out repetitive calculations of discretized PDEs until a solution is found [21]. Multi-step schemes include the two-step Lax-Wendroff method and relaxation methods like the Gauss-Seidel method [18]. We concentrate on single-step schemes for this study since applying multi-step schemes in an algorithmic pattern is not easy to automate. In addition, using multi-step schemes involves a heuristic process and may need user intervention to arrive at a solution.

**Table 1:** Finite difference scheme examples for the one-dimensional membrane potential propagation equation.

Scheme	Discretization for 1D Reaction-Diffusion Equation
	$$\frac{\partial v}{\partial t}=f\left(v\right)+D\frac{\partial^{2}v}{\partial x^{2}}$$
**FTCS**	$\frac{v_{n+1,j}-v_{n,j}}{\Delta t}=f\left(v_{n,j}\right)+D\frac{v_{n,j+1}-2v_{n,j}+v_{n,j-1}}{{\left(\Delta x\right)}^{2}}$
**BTCS**	$\frac{v_{n+1,j}-v_{n,j}}{\Delta t}=f\left(v_{n,j}\right)+D\frac{v_{n+1,j+1}-2v_{n+1,j}+v_{n+1,j-1}}{{\left(\Delta x\right)}^{2}}$
**Crank-Nicolson**	$\frac{v_{n+1,j}-v_{n,j}}{\Delta t}=f\left(v_{n,j}\right)+D\frac{v_{n,j+1}-2v_{n,j}+v_{n,j-1}}{2{\left(\Delta x\right)}^{2}}+D\frac{v_{n+1,j+1}-2v_{n+1,j}+v_{n+1,j-1}}{2{\left(\Delta x\right)}^{2}}$

To illustrate a single-step scheme, let us consider the monodomain model expression in Eq (1) for one-dimensional space. The resulting transformed equation is the membrane potential propagation equation:

$$\begin{array}{c} \frac{\partial V}{\partial t}=D\frac{\partial^{2}V}{\partial x^{2}}-\frac{I+J}{C_{m}}, \end{array}$$ (2)

where *x* is the independent spatial variable. The single-step discretization involves the replacement of differential operators and differential and arithmetic variables with their corresponding discrete terms. The differential and arithmetic variable replacement is given by,

$$\begin{array}{c} v\to v_{i_{1},i_{2},\ldots ,i_{n_{d}}} \end{array}$$ (3)

$$\begin{array}{c} y\to y_{i_{1},i_{2},\ldots ,i_{n_{d}}} \end{array}$$ (4)

where ***v*** is the set of all differential variables, ***y*** is the arithmetic variable set, and {*i*_1_, *i*_2_, …, *i*_*n*_*d*__} is the list of discrete time and spatial indices. The replacement of the differential operators depends on the specified discretization scheme. Each scheme presents a discretization for differential operators with respect to time and differential operators with respect to space. Table 1 shows some of the most common single-step schemes used in the FDM and their discretization for the one-dimensional membrane potential propagation equation terms. One of the simplest and easiest to derive is the explicit FTCS scheme. If we use the discrete terms for the FTCS scheme to discretize the membrane potential propagation equation in Eq (2) we get,

$$\begin{array}{c} \frac{V_{n+1,j}-V_{n,j}}{\Delta t}=D\frac{V_{n,j+1}-2V_{n,j}+V_{n,j-1}}{{\left(\Delta x\right)}^{2}}-\frac{I_{n,j}+J_{n,j}}{C_{m}}, \end{array}$$ (5)

where the index terms *n* and *j* are used for the time and space dimension, respectively. The FTCS scheme executes quickly but is impractical for most simulations due to its unstable nature. It is used in the majority of examples in this study to provide clarity of discourse. However, alternative methods with more general applicability can definitely be used with the code generator. Single-step finite difference schemes such as the fully-implicit Backward-Time Centered-Space (BTCS) method or Crank-Nicolson method offer more numerical stability.

Our study will be limited to the code generation of biological simulations using single-step FDM such as the ones described in Table 1. It includes the handling of both explicit schemes like FTCS and implicit methods like BTCS and Crank-Nicolson. The system also has the capability to handle the presence of simultaneous or nonlinear systems of equations in biological function models.

In addition to biological function model equations, our system can automatically handle a set of additional constraints, called boundary conditions. These are the conditions imposed on the boundaries of the simulation morphology. The effect of boundary conditions on the simulation is hard to overemphasize; it can mean the difference between a successful and an unsuccessful computation, or between a fast and a slow one.

One difficulty in handling boundary conditions comes from discretization. This is because the number of boundary conditions required by a finite difference formula depends on its stencil, not on the equation being modelled. Stencils are the geometric arrangements of a node group that relate to the interest point [22]. For example, the FTCS scheme uses a three-point stencil in one dimension, the interest point and the two neighbouring nodes in the *x* ^+^- (right) and *x* ^−^-direction (left). During discretization, a case may arise in which some stencil nodes are in a computational domain where the mathematical problem has no boundary and needs additional boundary equations. Our system handles this by ensuring that each unknown variable, including those in boundary points, has a corresponding equation to calculate for its value. This one-to-one correspondence of variables and equations ensures that the resulting computation arrives at a solution.

### Multidimensional Simulation Code Generation System

The main goal of our system is to generate program code automatically for multidimensional simulations involving PDEs with FDM. This is an extension of the system we published earlier to generate biological simulation code using ODE solving schemes [23]. In addition to the handling of nonlinear systems of equations, the current system includes the extensions from another study, which implements handling of multiphysics biological simulation [24].

**Fig 1:**
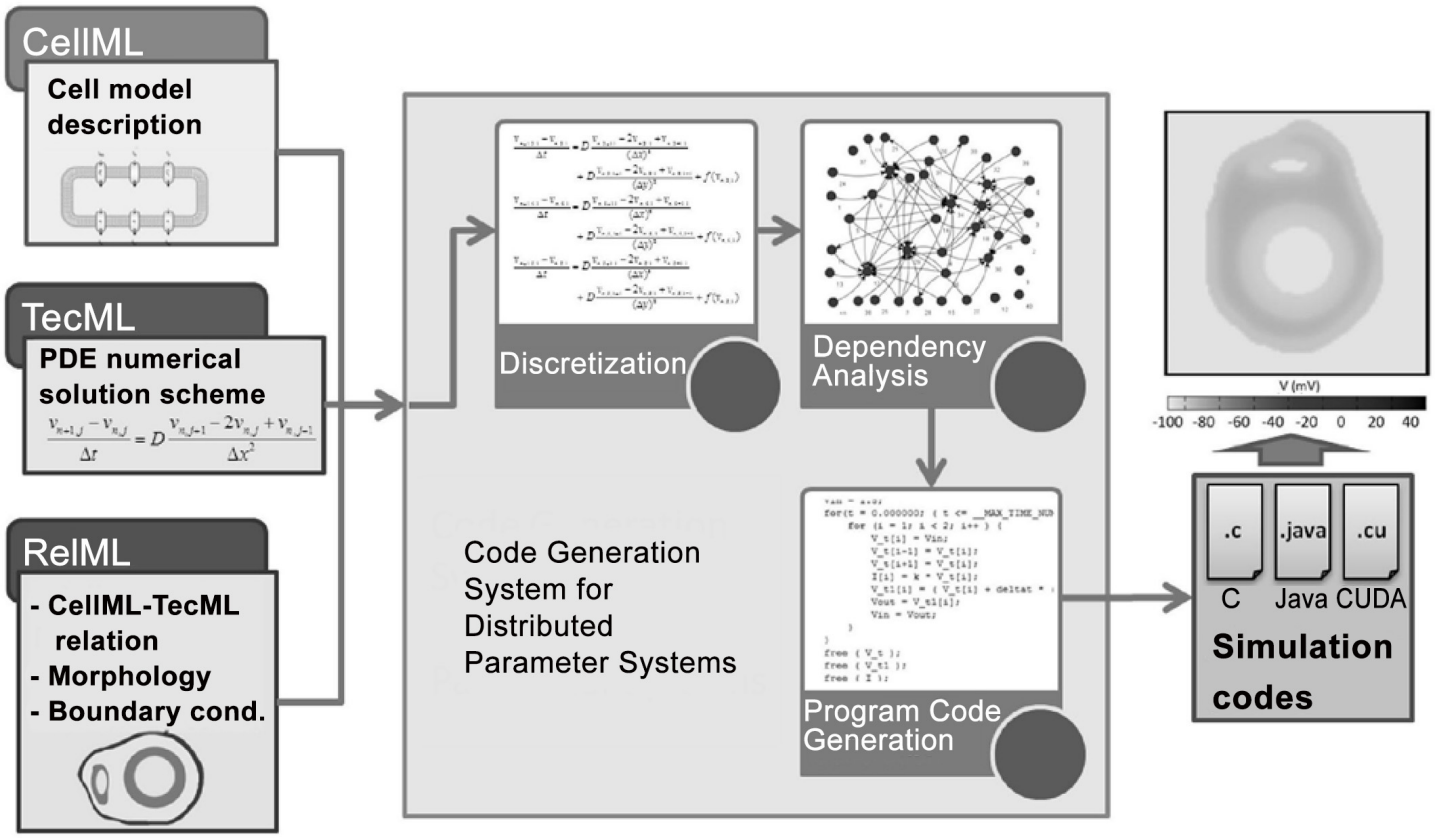
Overview of the proposed code generation system for distributed parameter systems with PDEs. The inputs include the CellML, TecML, and RelML files, while the output is a program code in C, Java, or CUDA programming language.

The system is composed of three stages: single-step PDE discretization and instantiation, dependency analysis, and program code generation (Fig 1). The discretization phase handles the inputs of the system and does the conversion of model equations and boundary conditions to their discrete equivalent. The resulting discrete model and boundary equations are then instantiated for each node in the morphology. Once all the equations are generated, the ordering is determined by the dependency analysis. Finally, code generation creates the program that will compute the simulation.

#### PDE discretization and instantiation

**Fig 2:**
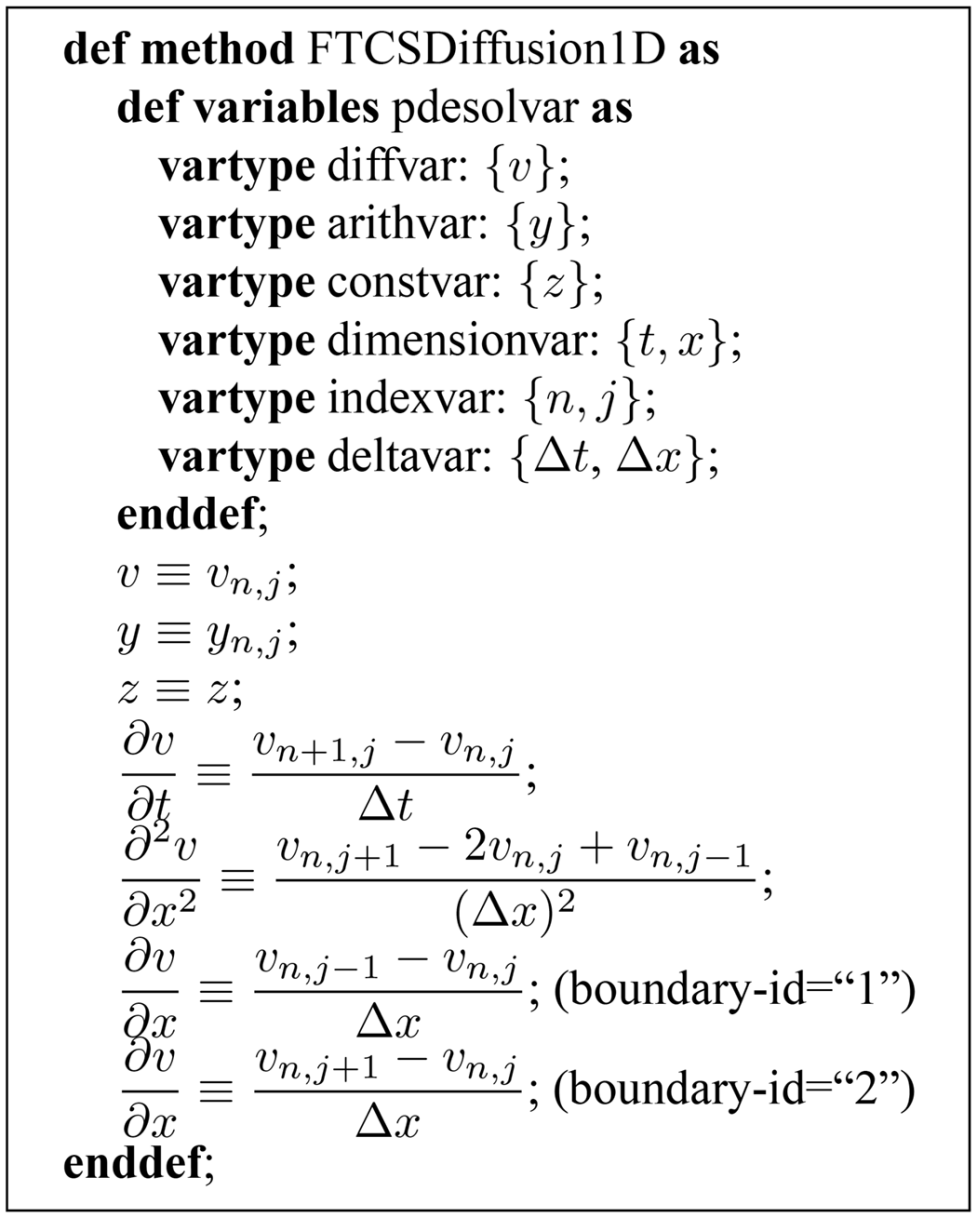
The TecML information for the FTCS method in one dimension. The first part lists all the variables and their types, while the second part enumerates the variable and operator discretization equations. The last two equations indicate the discretization of the Neumann boundary condition at both boundary points.

**Fig 3:**
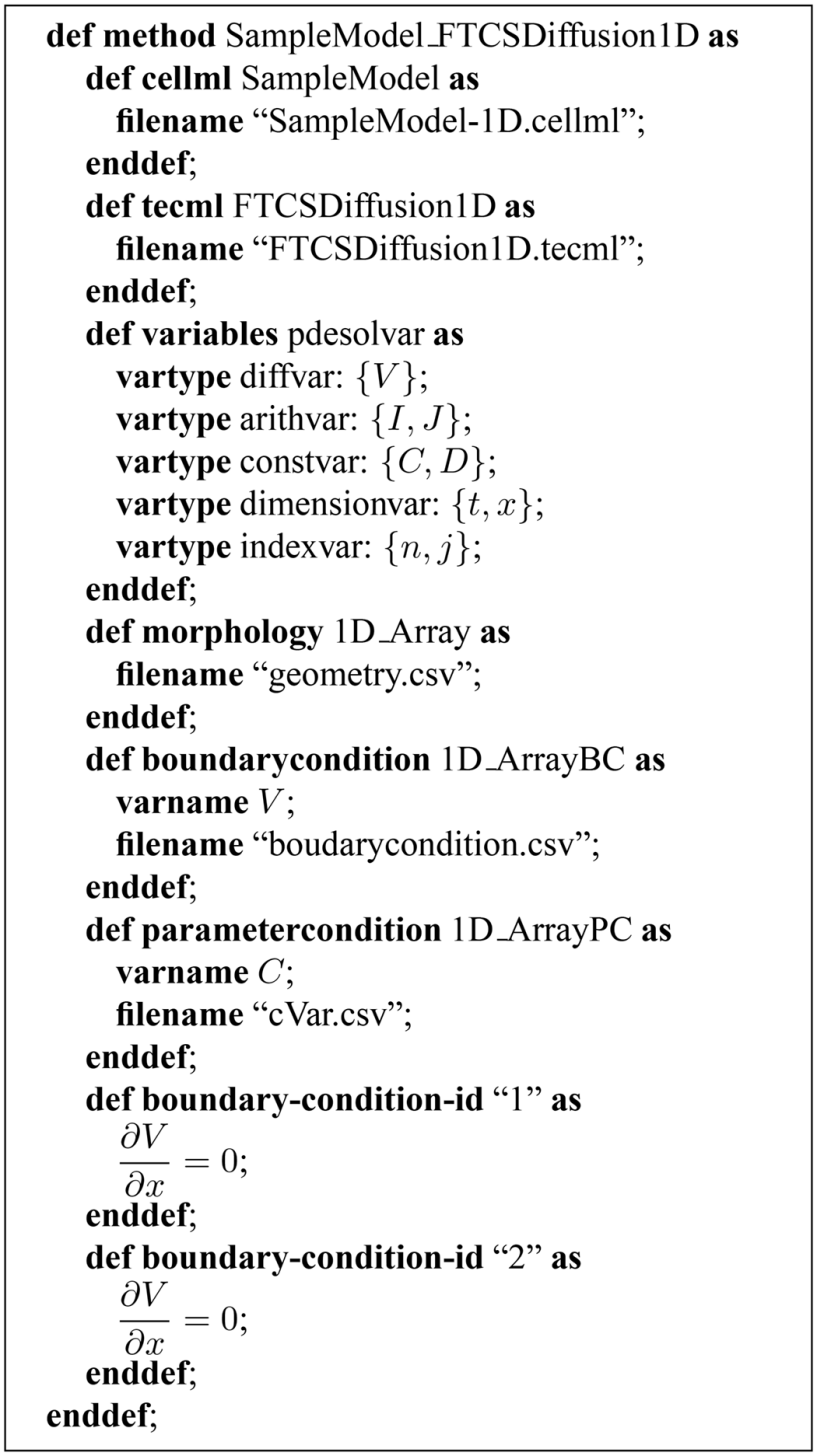
Information contained in the RelML file for a simulation involving a simple model with a membrane potential propagation partial differential equation and the FTCS as the numerical solution scheme. The first part describes the relation between variables in the two files. The spatial geometry, spatial boundary condition, and actual boundary condition information are also included.

The inputs needed to generate a simulation program are: a CellML or PHML file describing the biological function model; a TecML (Time Evolution Calculation Markup Language) file detailing the FDM scheme (Fig 2); and a RelML (Relation Markup Language) file indicating the relation between the first two inputs (Fig 3) [13]. CellML is an open standard based on extensible markup language (XML) and used to store and exchange computer-based mathematical models. PHML or Physiological Hierarchy Markup Language can describe multilevel structures of physiological functions in mathematical models. Both languages are used to describe biological function models used in cardiac electrophysiology simulation.

In our previous studies, we introduced the XML-based TecML file format and used it to describe ODE numerical solution schemes [23]. This was extended to include the capability to describe finite difference methods for solving PDEs [13]. As mentioned in our previous study, we used RelML to describe the relationship between a CellML or PHML file and TecML file. RelML maps the variables in the biological function model file to their corresponding type in the numerical solution scheme file. It also contains information about the morphology and boundary conditions of the underlying PDEs. A new RelML format was used for the simulations in this study and the details of how RelML was used during discretization was described in the algorithm part of this section. The descritization algorithm was not detailed in our previous studies.

The TecML file specifies the replacement pattern for each type of variable and differential operator [13]. It lists the symbol for each of the predefined variable types and their corresponding discrete terms. The variable types include differential variables, arithmetic variables, and constants. In addition, the TecML file gives the discretization term for the nth-order differential with respect to the time or spatial variables.

**Fig 4:**
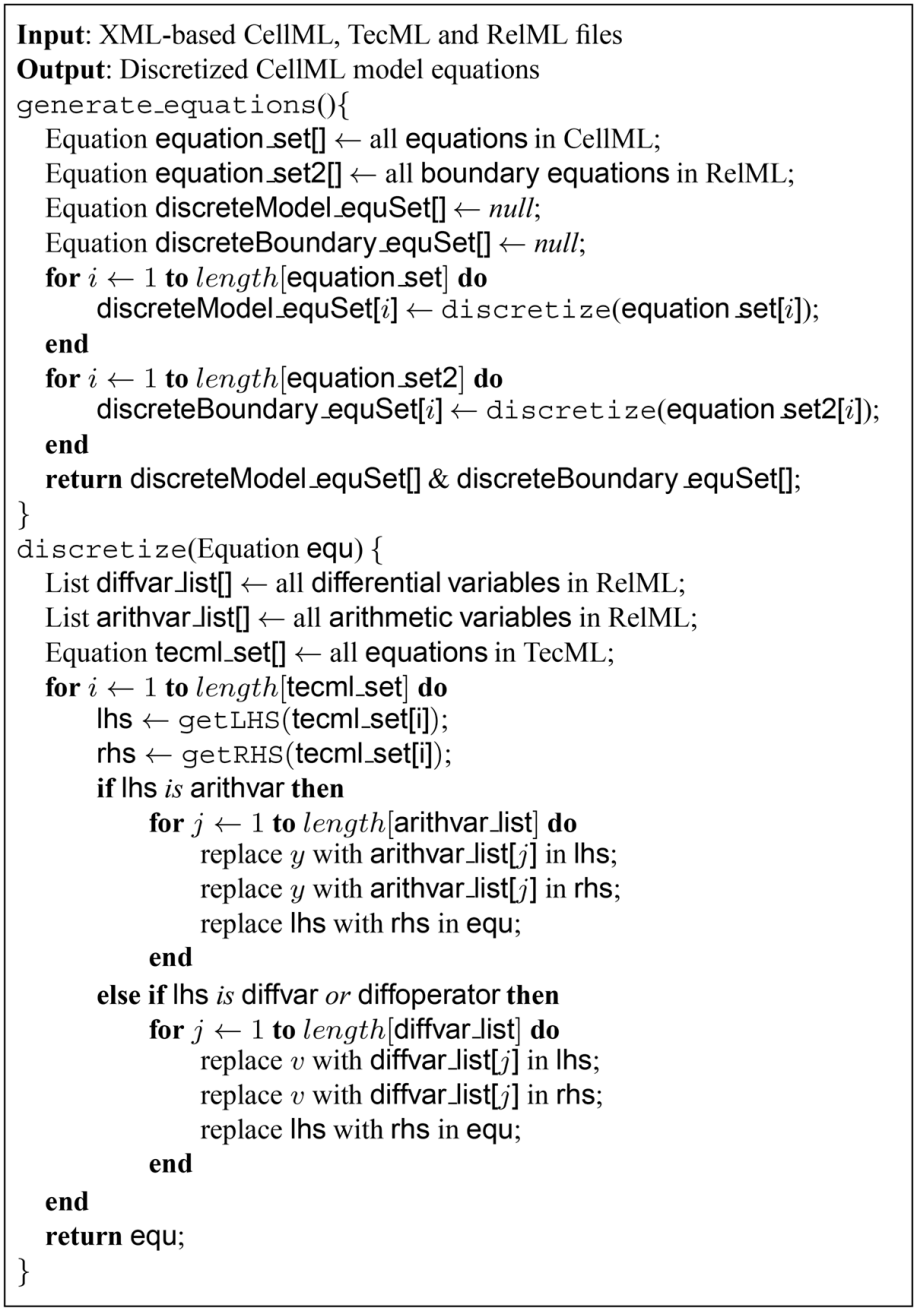
Algorithm for generating the set of discrete PDE numerical solutions and boundary condition equations.

During discretization, the algorithm uses the replacement patterns in TecML to discretize each and every equation in the biological function model. For each equation in the model, the algorithm takes every variable, gets its corresponding variable type specified in the RelML file, and checks for the replacement pattern of that variable type in the TecML file. A replacement pattern is found if the variable type is in the left-hand side (LHS) of one of the discretization equations. If a pattern exists, the algorithm proceeds to replace that variable type with the model variable in the right-hand side (RHS) of the discretization equation. Finally, the resulting discrete term from the RHS is used to replace the original variable in the biological function model. The actual variable names used in the model are retained during discretization. The algorithm in Fig 4 details the steps for discretizing the model equations and boundary conditions using the PDE numerical solution scheme described in TecML.

A sample TecML file for the FTCS scheme in one dimension is shown in Fig 2. It lists the identifier for the predefined variable types, which include the differential variable (*diffvar: v*), arithmetic variable (*arithvar: y*), and constants (*constvar: z*). The variable (*dimensionvar: t, x*) and index (*indexvar: n, j*) corresponding to each dimension and the unit change per dimension (*deltavar: Δ*t*, Δ*x**) are in the list as well. It also describes the discretization of the different types of boundary conditions that may appear in the RelML file. Note that compact syntax representation is used to show the contents of TecML in Fig 2 and is not the actual file. This notation is based on Alan Garny’s notation for CellML in the Cellular Open Resource (COR) software [25].

The discretization of the biological function model equations follows a distinct order with respect to the variable types. Although the discretization of the equations can be in any order, the order of discretizion in each equation is as follows: *arithvar* first, then *diffvar*, and differential operators last. This replacement procedure is represented in Strachey brackets and expressed by the following function:

$$\begin{array}{c} R_{f}⟦equ⟧=R_{o}⟦R_{v}⟦R_{y}⟦equ⟧⟧⟧, \end{array}$$ (6)

where ⟦*equ*⟧ is a model equation. **𝓡**_***f***_⟦*equ*⟧ is the final discretized equation and **𝓡**_***o***_, **𝓡**_***v***_ and **𝓡**_***y***_ correspond to the differential operator, differential variable, and arithmetic variable replacement, respectively.

To illustrate the steps in how the equations in a biological function model are discretized using TecML, consider the following set of model equations:

$$\begin{array}{c} \frac{\partial V}{\partial t}=-I, \end{array}$$ (7)

$$\begin{array}{c} I=J+D\frac{\partial^{2}V}{\partial x^{2}}, \end{array}$$ (8)

$$\begin{array}{c} J=\{\begin{array}{cc} C & \text{if}\;0\leq t\leq 10\\ 0 & \text{otherwise}\; \end{array}, \end{array}$$ (9)

where *D* is the diffusion constant, and *C* is a location-dependent variable. A first-order partial differential with respect to time is applied to *V* and a second-order differential is applied with respect to the spatial direction *x*. The first two equations combined represent a membrane potential propagation system. In this example, *V* corresponds to variable type *diffvar* whereas *I* and *J* are categorized as *arithvar*. Constants *D* and *C* are considered as *constvar*. This mapping information is provided in the RelML file, which links the biological function model and TecML file. Variable *C* is considered a constant but is a distributed parameter variable. This means that its value varies according to the location of the material or cell node. Our system can handle these location-dependent variables by allowing users to supply a list containing the distributed parameter value at each coordinate point in the simulation morphology and supply that information through RelML. Here, if the user wants to assign the value of *C* only at the first node, he or she needs to supply the value of *C* at node *j* = 1 and set the rest to zero. Fig 3 shows the information in the RelML file, including the geometry, distributed parameters, and boundary condition equations.

The resulting discretization of Eqs (7)–(9) is given by,

$$\begin{array}{c} \frac{V_{n+1,j}-V_{n,j}}{\Delta t}=-I_{n,j} \end{array}$$ (10)

$$\begin{array}{c} I_{n,j}=J_{n,j}+D\frac{V_{n,j+1}-2V_{n,j}+V_{n,j-1}}{{\left(\Delta x\right)}^{2}}, \end{array}$$ (11)

$$\begin{array}{c} J_{n,j}=\{\begin{array}{cc} C_{j} & \text{if}\;0\leq t\leq 10\\ 0 & \text{otherwise}\; \end{array}, \end{array}$$ (12)

where *n* and *j* are the temporal and spatial index, respectively. Given the constants, the value of *V* at *n* = 0, and *C*_*j*_ as a distributed constant, the equation set has three equations and three unknowns.

Once the biological function model and boundary conditions are discretized, they are instantiated to generate the input for the dependency analysis. The instantiation uses the information provided in the geometry node list. The node list contains all the coordinates in the one-dimensional, two-dimensional, or three-dimensional mesh, which contains the geometry to be used for the simulation. All discrete model equations are instantiated for each node in the geometry by replacing the spatial index of the equations with the node coordinates. This also applies to the boundary condition where the discrete boundary equations are instantiated at the geometry boundary points. The generation of all equations for geometry node was done to achieve generality in handling different numerical solution schemes and boundary conditions.

The geometry, boundary condition, and distributed parameters information provided in a RelML file (Fig 3) are described as follows:

1. The geometry node list is supplied as a list of three-dimensional coordinates and their corresponding morphology value. Each morphology value is either “0” or “1”. A value of “1” indicates that the coordinate point is part of the simulation morphology, while a “0” means it is part of the background or empty space.
2. Boundary nodes refer to the background nodes that are in proximity of morphology nodes. The boundary node list is supplied with each of the node’s coordinates and a boundary condition type identification number. This number indicates the position of the boundary node with respect to the morphology node of interest (in the case of a three-point stencil, whether it is on the left or right) and if it is either a Dirichlet or a Neumann condition.
3. A distributed parameter is a variable that varies in value depending on morphology location. Each distributed parameter variable has a designated file that contains the list of all the mesh nodes and the parameter value at each node.

These information are written in individual CSV (comma-separated values) files and are needed to provide the system with the necessary data during the instantiation process. The output of the instantiation process serves as the input for the dependency analysis phase.

#### Dependency analysis

In modelling, it is necessary to identify the dependency between variables and the structure of model equations. This structure, along with data dependence, affects how the resulting program code should be organized. Dependency analysis is important since it confirms whether boundary conditions are applied to the model equations and all the unknown variables can be calculated. This is done by ensuring that there is a corresponding equation for each unknown variable for all the nodes in the simulation morphology.

Our system automatically handles the dependency analysis of the model equations and accompanying boundary conditions. Dependency analysis is performed on the instantiated equation set, in which the spatial indices are replaced with coordinate location values. First, a maximum matching method [26] is used to determine the correspondence between each unknown variable and the equation to calculate that variable. Maximum matching starts with a bipartite graph *G* = (*A*∪*B*, *E*), where *A* is the set of unknown variables, *B* is the set of equations and *E* denotes the edges of the graph. The edges in *G* are 2-element links between between a variable and an equation vertex. A bipartite graph is a graph whose vertices are divided into two disjoint sets [27]. In this case, the unknown variables and the equations are the two sets of the bipartite graph. A perfect matching *S* ⊂ *A* × *B* is searched to match every unknown variable vertex to an equation vertex in graph *G*. In the case of simultaneous equations, each equation in the simultaneous equation set is matched to the variable in its left-hand side. This assumes that there is at least one unknown variable in the left side of each equation. A perfect matching happens if each variable is matched to one and only one equation and vice versa. For the matching algorithm, the user has the option of using either the Ford-Fulkerson [28] or Hopcroft-Karp [29] algorithm.

After perfect matching of the bipartite graph is achieved, the system then tries to get the computation order of the equations using Tarjan’s algorithm [30]. To get the computation order, a topological ordering of the directed graph from the perfect matching is needed. A topological ordering is possible if and only if the graph has no directed cycles. Tarjan’s algorithm is a procedure for finding strongly connected components or cycles in a directed graph. The algorithm combines the nodes of a directed cycle into a single node, hence creating a graph without directed cycles. Here, the components are equations and strongly connected equations exist if (a) the model contains simultaneous equations or (b) an implicit finite difference method is used or both.

#### Program code generation

Once all the instantiated equations are sorted, they are constructed into loops. A loop is created whenever there is a series of instantiated equations from consecutive nodes. Let us take Eq (10) as an example and assume that the unknown variable is isolated in the left hand side of the equation. If the equation is instantiated for three consecutive nodes numbered 1–3, the sorted equations are given by

$$\begin{array}{c} V_{n+1,1}=V_{n,1}+\Delta t\left(-I_{n,1}\right), \end{array}$$ (13)

$$\begin{array}{c} V_{n+1,2}=V_{n,2}+\Delta t\left(-I_{n,2}\right), \end{array}$$ (14)

$$\begin{array}{c} V_{n+1,3}=V_{n,3}+\Delta t\left(-I_{n,3}\right). \end{array}$$ (15)

The loop construction process will take the three equations above and combine them into a single equation iterated over spatial nodes 1–3, as shown by the pseudocode line

$$\begin{array}{c} \text{for(}i=1\;\text{to}\;3)\;V_{n+1,i}=V_{n,i}+\Delta t\left(-I_{n,i}\right). \end{array}$$ (16)

Equations that are iterated over the same chain of consecutive nodes are put together in the same loop. There are instances, however, where two or more loops iterating over the same nodes are created. This happens if groups of equations are sorted as independent from each other and put into separate loops. Loops iterated over boundary points are also constructed for the boundary equations. All the spatial loops are inserted into the main time loop, where the equations are iterated over the simulation time. The ordering of the spatial loops inside the time loop is determined by the dependency analysis results.

If there are simultaneous equations inside a spatial loop, a Newton solver with a Jacobian function for solving nonlinear systems of equations is added to the generated code. A function call for a Newton solver is called inside the loop with the simultaneous equations and list of unknown variables as parameters.

In addition to the creation of loops, the code generator automatically inserts the declarations of variables and headers needed to compile and run the program. For the C program generation, memory allocation of the variable arrays is also inserted. The resulting C and Java source code can be compiled by commonly used C and Java compilers, respectively.

The code generator software itself is written in Java. The source code of the software is available and can be downloaded online [31].

### Boundary Condition Handling

The system handles the implementation and discretization of the boundary condition during the discretization phase. Boundary condition equations are read from the RelML file and discretized using the numerical scheme described in the TecML file. The system can implement the two most commonly used boundary conditions for solving PDEs: Dirichlet and Neumann boundary conditions. A Dirichlet boundary condition specifies the value of the function on a point, line, or surface in 1D, 2D or 3D, respectively. For the membrane potential propagation example in Eq (2), a Dirichlet condition can be given at the start and end points of the simulation array with

$$\begin{array}{c} V(0)=a,V(L)=b,\;0\leq x\leq L. \end{array}$$ (17)

A Neumann boundary condition, on the other hand, specifies the values that the derivative of a solution takes on the boundary of the domain. In the one-dimensional example, the Neumann boundary conditions can be

$$\begin{array}{c} {\frac{\partial V}{\partial x}|}_{x=0}=0,\;{\frac{\partial V}{\partial x}|}_{x=L}=0. \end{array}$$ (18)

The zero Neumann boundary condition indicates that the charge is contained inside the simulation morphology (no-flux boundary condition). The RelML file specifies the condition on each defined boundary (*x* = 0;*x* = *L*) since the discretization for each boundary might be different. The TecML file then needs to give the discretisation for each boundary condition in the RelML file as shown by the following equation pairs:

$$\begin{array}{c} \frac{\partial V^{-}}{\partial x}=0\to \frac{V_{n,j-1}-v_{n,j}}{\Delta x}=0, \end{array}$$ (19)

$$\begin{array}{c} \frac{\partial V^{+}}{\partial x}=0\to \frac{V_{n,j}-v_{n,j+1}}{\Delta x}=0. \end{array}$$ (20)

where Eqs (19) and (20) indicate the discretization for the left (*x* = 0) and right (*x* = *L*) boundary point, respectively.

In addition to using either of the two mentioned boundary conditions, the system can also implement a mixture of the two types. This is commonly referred to as a mixed boundary condition. The user can also separate the boundary domain into any number of parts and specify what type of boundary condition to use in each part. The system, however, cannot handle a Robin boundary condition, which is a linear combination of Dirichlet and Neumann boundary conditions.

Once the discrete boundary conditions are generated, the equations are instantiated based on the CSV file containing the boundary identification number (ID) for each boundary node in the morphology. Each boundary node has a boundary ID and an instance for that node is generated from the corresponding boundary condition type and discrete boundary equation. A boundary ID corresponds to either a *left* or *right* direction discretization as shown in Eqs (19) and (20). The complete list of possible boundary discretizations and their corresponding boundary IDs is written in a TecML file (Fig 2). In the case of mixed boundary conditions, the IDs can be used to enumerate all the parts of a boundary domain and their condition type.

## Results and Discussion

To validate automatic handling of PDE boundary conditions in our code generation system, we created two sets of experiments. The first set of experiments tested the capability of our system to automatically adjust the calculation order of the simulation equations depending on the boundary condition and numerical solution scheme used. The second set of experiments involved the generation of program codes for cardiac electrophysiology simulations and comparison of the simulation results with published experimental data.

### Boundary Conditions and Dependency Analysis Results

**Fig 5:**
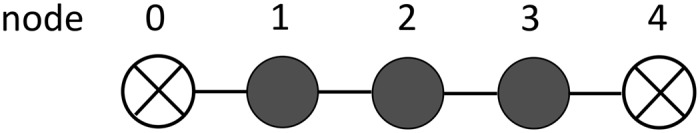
The one-dimensional morphology example with three material nodes bounded by two empty nodes.

To illustrate the effect of using different boundary conditions on the dependency analysis result, we applied Dirichlet and Neumann boundary conditions to the model given by Eqs (7)–(9). A single dimension array with three morphology nodes and two boundary nodes, shown in Fig 5, was used as the morphology mesh. Applying the instantiation process to previous example Eqs (10)–(12) and using the FTCS method, we generated nine instances of the model equations. If a Neumann boundary condition is used at both boundaries, two equations for solving the value of *V*_*n*,*j*_ at the left and right boundaries are added. These boundary equations are generated as instances of Eqs (19) and (20), where *j* = {1,3}, to get

$$\begin{array}{c} \frac{V_{n,0}-V_{n,1}}{\Delta x}=0, \end{array}$$ (21)

$$\begin{array}{c} \frac{V_{n,3}-V_{n,4}}{\Delta x}=0. \end{array}$$ (22)

**Fig 6:**
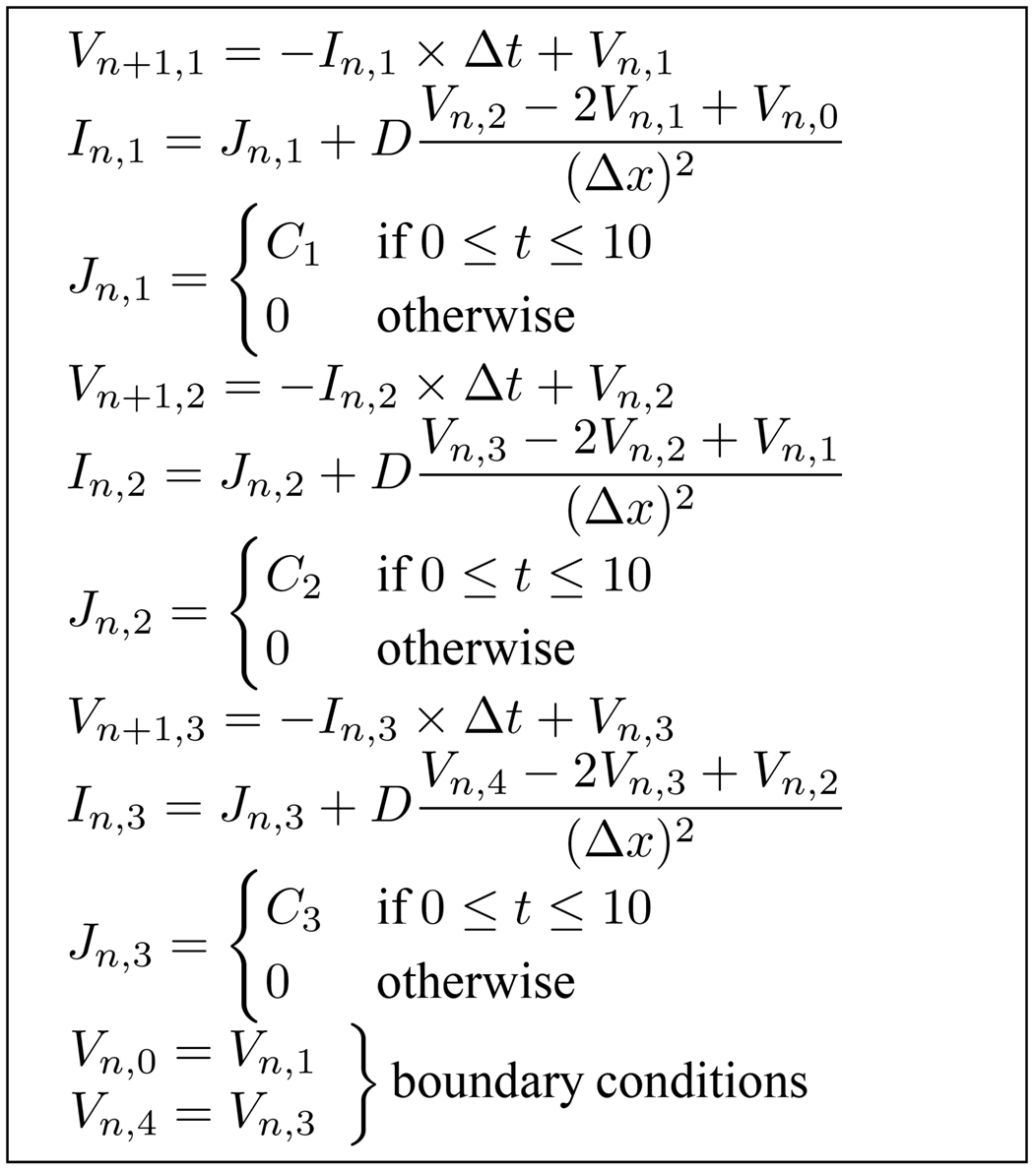
Generated instances of the example model equations with Neumann boundary conditions. The equations are arranged to show the unknown variable at the left hand side of each equation.

**Fig 7:**
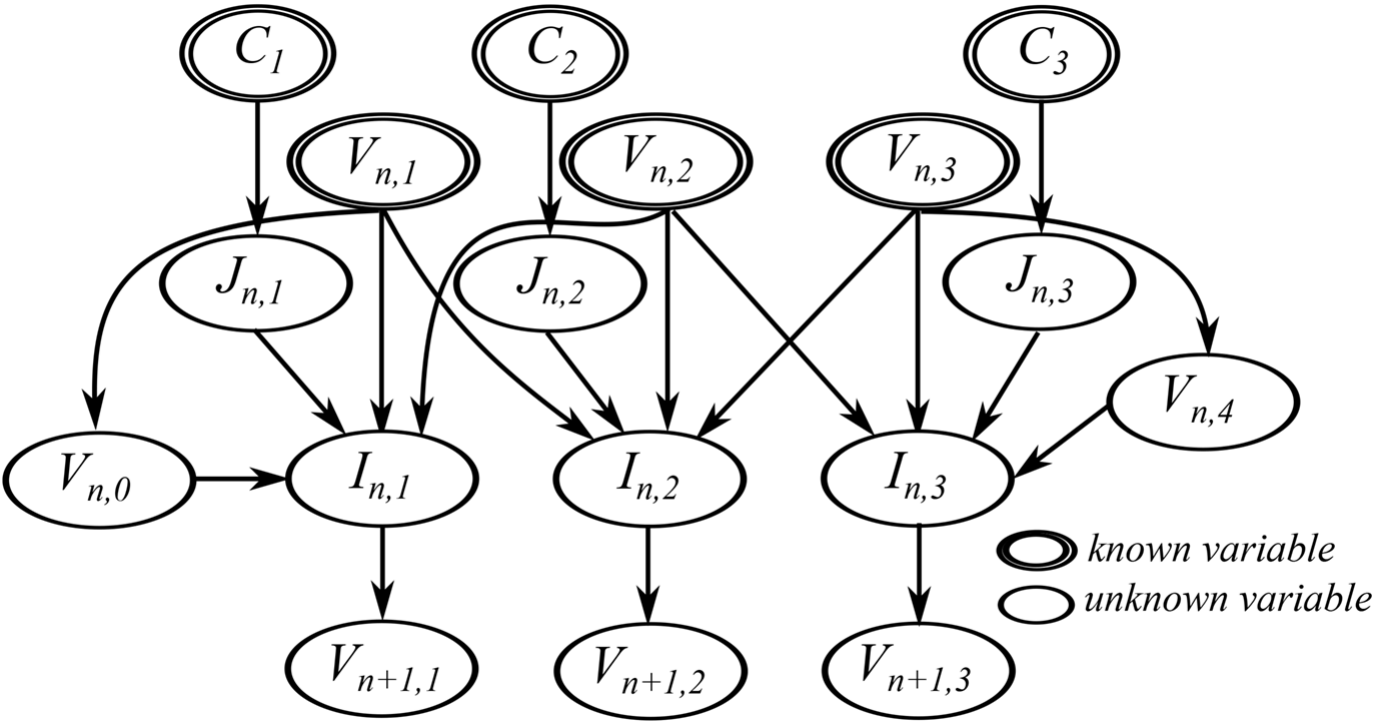
The resulting dependency graph for the sample equation set with Neumann boundary conditions.

This results in a set of 11 equations and 11 unknown variables, shown in Fig 6 with the unknown variable at the left hand side of each equation. Doing dependency analysis on this equation set results in the graph in Fig 7. Each node in the graph indicates the matched unknown variable and its corresponding equation in the variable-equation bipartite graph. For simplicity, the name of the unknown variable was used to represent each node. Note that the stimulation current, *J*, is treated as a distributed parameter variable.

Changing the boundary condition of the simulation alters the dependency analysis results. If we change from Neumann to Dirichlet boundary conditions, we will get the following equations:

$$\begin{array}{c} V_{n,1}=a, \end{array}$$ (23)

$$\begin{array}{c} V_{n,3}=b, \end{array}$$ (24)

where *a* and *b* are constants. Note that the boundary conditions apply at any point in time, therefore, *V*_*n*+1,1_ = *a* and *V*_*n*+1,3_ = *b* are also included in the equation set.

**Fig 8:**
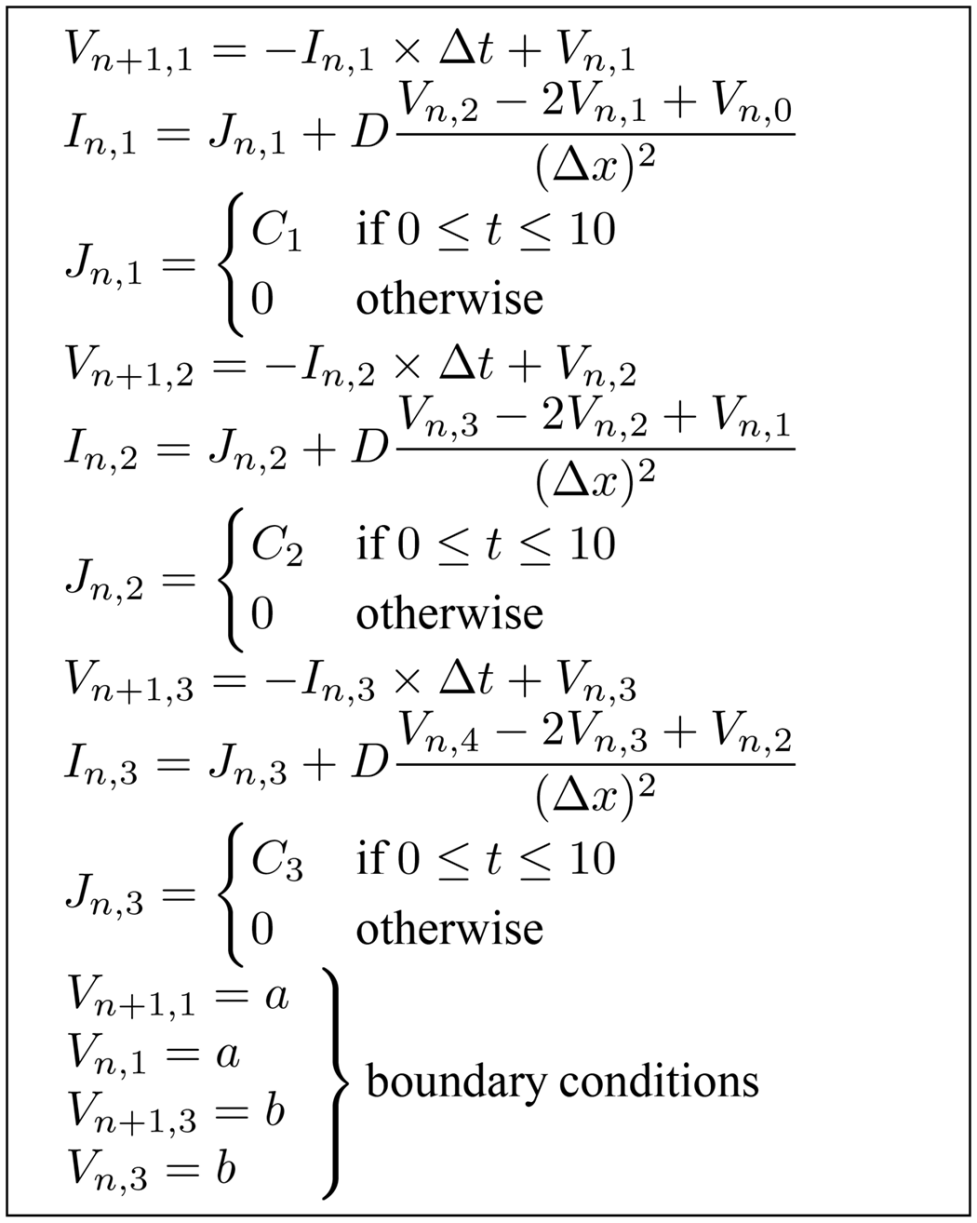
Generated instances of the example model equations with Dirichlet boundary conditions.

**Fig 9:**
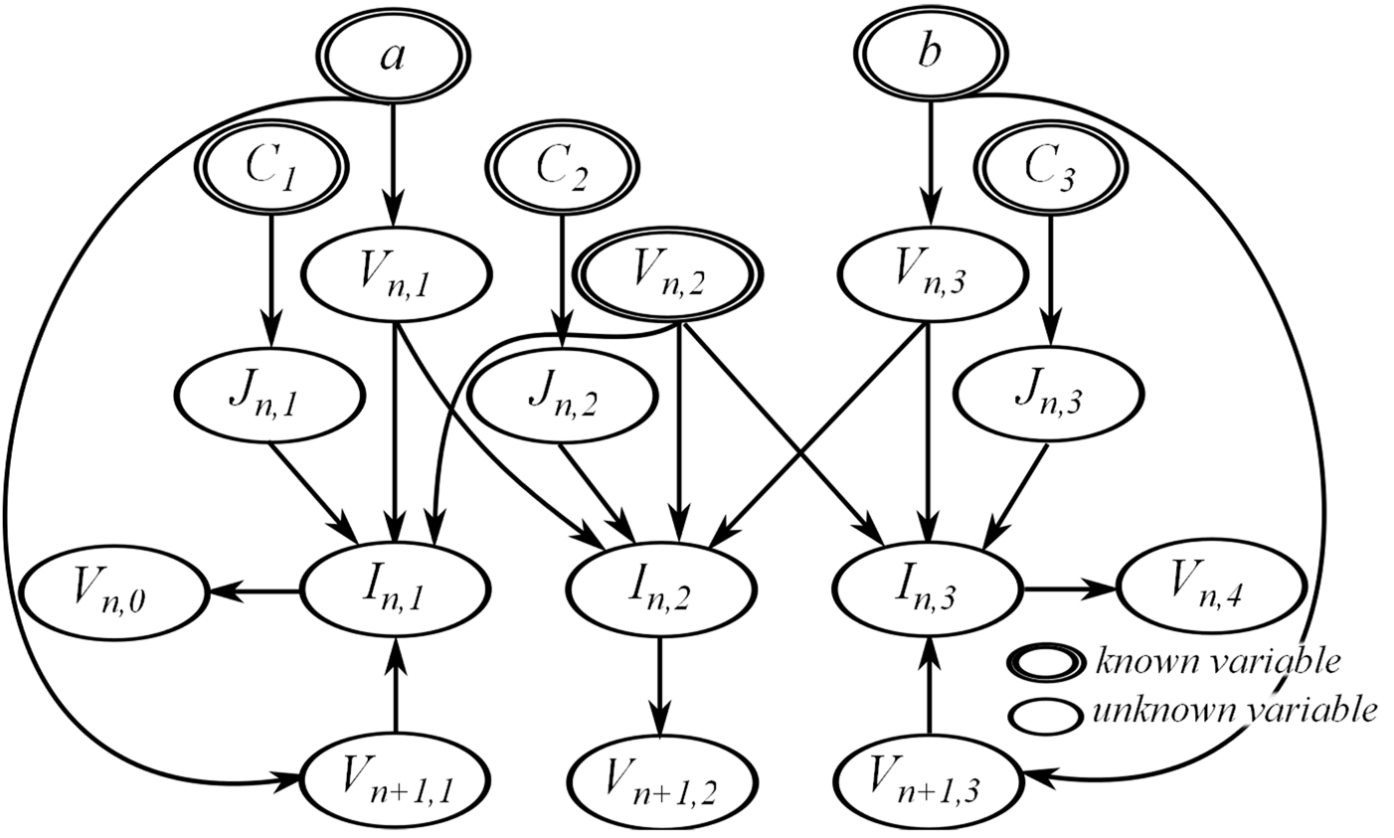
The resulting dependency graph for the sample equation set with Dirichlet boundary conditions.

The resulting equation set with Dirichlet boundary conditions has 13 equations with 13 unknowns (Fig 8). This set includes equations with constants assigned to the value of *V*_*n*+1,1_ and *V*_*n*+1,3_, making them source variables. On the other hand, the value of *V*_*n*+1,2_ is still derived from the model equations. This results in the dependency graph shown in Fig 9.

Comparing the two dependency graphs, it is apparent that the choice of boundary condition affects the number of unknown variables in the equation set. This leads to a different dependency graph, thus changing the calculation order and resulting program code. The arrows in the dependency graph for *j* = 1 changed direction in Fig 9. Since *V*_*n*+1,1_ is given from the Dirichlet boundary condition, the value of *I*_*n*,1_ is computed from it and the unknown variable *V*_*n*,0_ is computed from *I*_*n*,1_.

The type of discretization method used can also affect the dependency graph. If implicit methods are used, the dependency graph will contain simultaneous equations and require different boundary equations. Let us take Eqs (10)–(12) and use Dirichlet boundary conditions again as an example. If we use BTCS instead of FTCS to discretize the equations, there will be no need for the values of *V*_*n*,*j*_ at the boundaries. The required boundary equations are

$$\begin{array}{c} V_{n+1,1}=a, \end{array}$$ (25)

$$\begin{array}{c} V_{n+1,3}=b. \end{array}$$ (26)

However, we also need the values of *V*_*n*+1,0_ and *V*_*n*+1,4_ to solve *I*_*n*,1_ and *I*_*n*,3_, respectively. If we assign the values we gave to the boundaries to these unknowns, we get

$$\begin{array}{c} V_{n+1,0}=a, \end{array}$$ (27)

$$\begin{array}{c} V_{n+1,4}=b. \end{array}$$ (28)

**Fig 10:**
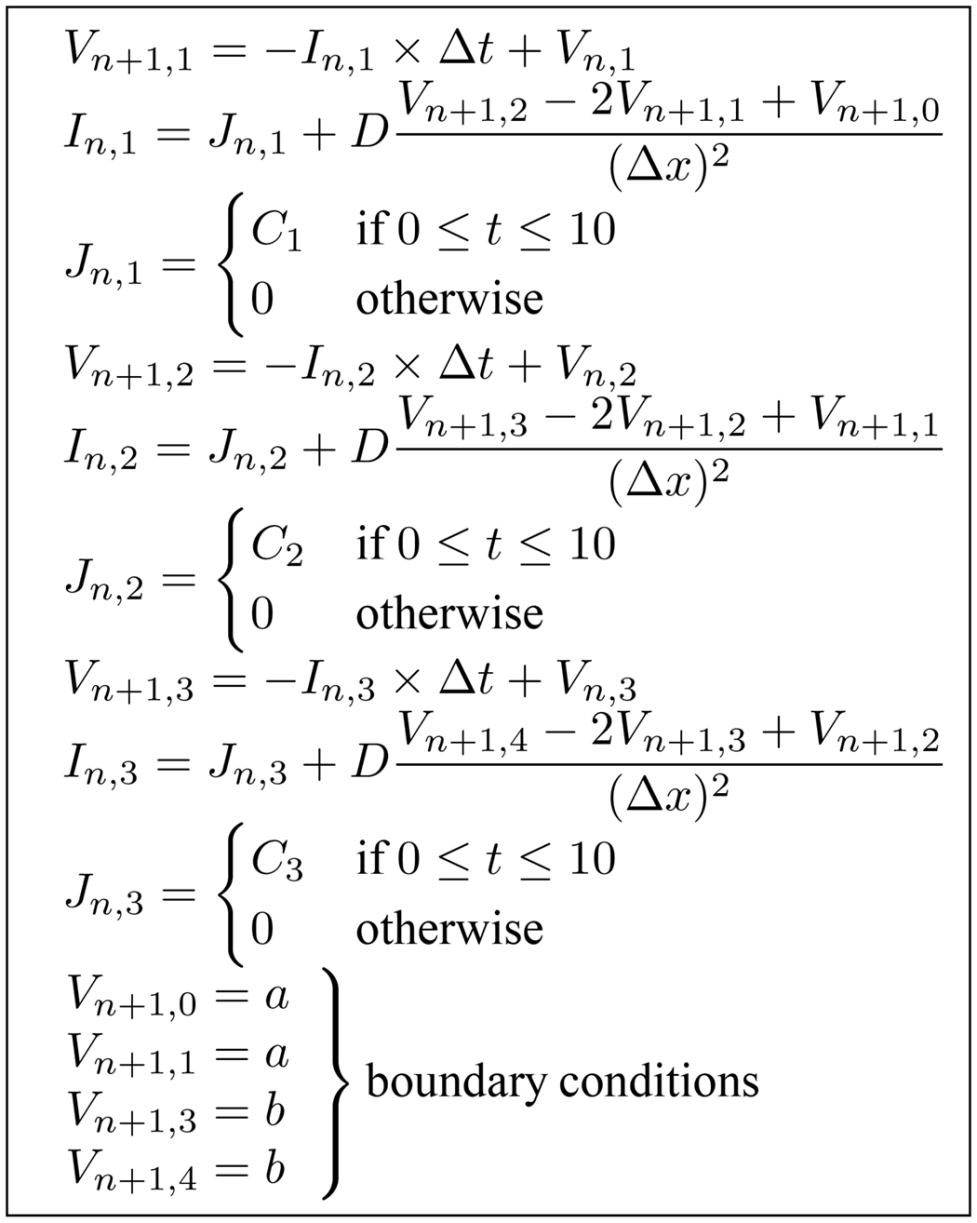
Generated instances of the example model equations with Dirichlet boundary conditions and the BTCS method used for discretization.

**Fig 11:**
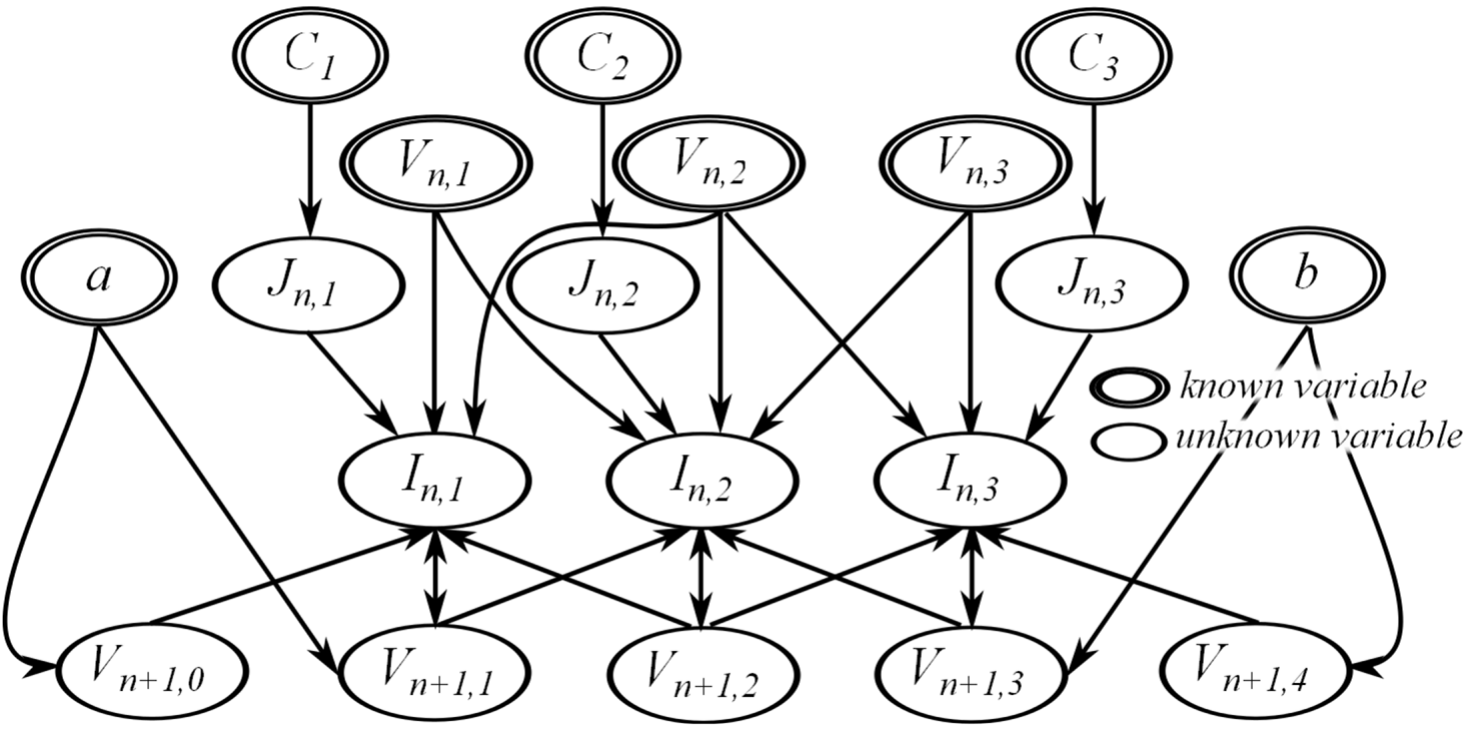
The resulting dependency graph for the sample equation set with Dirichlet boundary conditions and the BTCS method used for discretization.

The resulting equation list shown in Fig 10 now has simultaneous equations. Values of *I*_*n*,*j*_ are both used for calculations of and depend on *V*_*n*+1,*j*_, as shown in the bidirectional arrows in Fig 11.

Manually creating program code using complex biological function models or morphologies for the three different cases can result in entirely different programs and lead to longer coding times. Our system automates the handling of boundary conditions and can make it easier to test different boundary types for simulation.

### Code Generation Time

To measure the performance of our system, we measured the time it takes to generate program code for a couple of biological function models. The first model we used was the Luo-Rudy 1991 (LR1) model [32]. The LR1 model is a computational formula for the action potential of a guinea pig ventricular cell. It is composed of 39 equations, including eight differential equations. The other model that we used was the Hund-Rudy (HRD) model [33], which is based on canine ventricular cell data. The HRD model is composed of 134 equations, 29 of which are differential equations. The code generator runs on a Windows Server 2012 computer with a 64-bit operating system. The computer has an AMD Opteron processor and 32 gigabytes (GB) of random-access memory (RAM).

We generated program code for a number of mesh morphology sizes. All the morphologies are in two dimensions, uniform grid, and square-shaped. The mesh sizes used range from 20 × 20 to 100 × 100. Two types of FDM schemes were also used to measure the difference in code generation time between explicit and implicit schemes. The FTCS method is used for the explicit scheme, while the Crank-Nicolson method was the implicit scheme example. Mesh sizes of up to 100 × 100 were used to generate code for the FTCS method. However, due to the amount of time it takes to generate the Newton solver code for implicit methods, mesh sizes of only up to 40 × 40 were generated for the Crank-Nicolson method.

**Fig 12:**
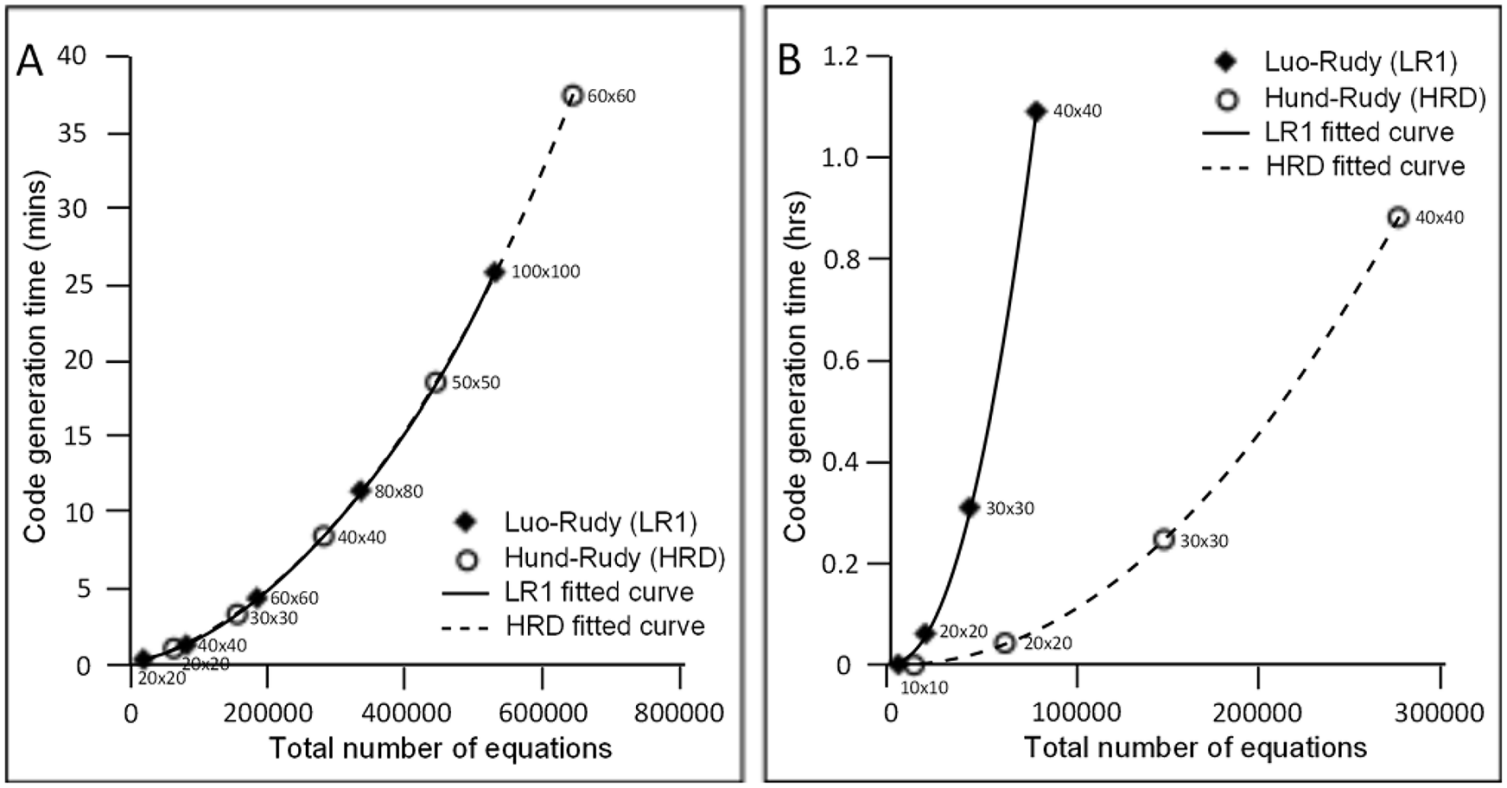
Measurement of code generation time for the Luo-Rudy (LR1) and Hund-Rudy (HRD) model using the explicit FTCS (A) and implicit Crank-Nicolson (B) method. The mesh sizes used for the FTCS method varied between the LR1 and HRD model. LR1 program code was generated using mesh sizes of up to 100 × 100, while HRD code generation used meshes of up to 60 × 60. For the Crank-Nicolson method, the same set of meshes is used for both models in generating code. The total number of equations is approximately equal to the number of equations in the model multiplied by the number of nodes in the mesh. Generation time of simulation code using explicit methods is in minutes while it takes hours to generate code using implicit methods.

The results of the code generation time measurements are shown in Fig 12. Generation time is plotted against the total number of equations, which depends on the biological function model and mesh size. The total number of equations in each plot point is proportional to the number of nodes in the mesh and the size of the model. The FTCS method was used for the results in Fig 12A, while results in Fig 12B are for the Crank-Nicolson method. The measured code generation time for a simulation with 646,122 equations and using the explicit FTCS method was approximately 38 minutes. Code generation using the implicit Crank-Nicolson method, however, took much longer. Generation time for a simulation with 79,728 equations took more than an hour. This was due to the time it takes to generate the Newton solver function, which takes more than 99% of the total code generation time. Generating the Newton solver function involves getting the *k*-by-*k* Jacobian matrix, where *k* is the total number of simultaneous equations.

### Cell-to-Cell Coupling Simulation

Program codes were generated for cell-to-cell coupling simulations using Dirichlet and Neumann boundary condition in separate experiments. The simulation programs were generated code in C programming language. The programs were compiled using Cygwin C Compiler and were run in a Windows 7 computer with an Intel Core i7 880 processor and 8 GB of memory.

The first set of simulations was done to measure the decay of subthreshold transmembrane potential (*V*_*m*_) in space. Akar et al. [34] published an experimental study on the measurement of cell-to-cell coupling in an intact heart using subthreshold stimulation. A subthreshold stimulus refers to a stimulus that activates a transduction process but is not enough to fire an action potential.

The published study was based on assumptions from cable theory, which is the mathematical analysis of signal propagation through space, for instance, through spatially extended nerve or cardiac cells. The experiment made optical measurements of *V*_*m*_ from a mapping region in an adult guinea pig heart. A stimulating electrode was placed in the center of the region and measurements were recorded from sites 0–2.5 mm away from the stimulating electrode. To plot the membrane potential decay curve, they expressed the value of *V*_*m*_ as the ratio of the difference between the membrane potential and its highest value with the difference between the highest and lowest value ((*V*_*max*_ − *V*)/(*V*_*max*_ − *V*_*min*_)).

Three different models were used—namely, the FitzHugh-Nagumo (FHN) [35], LR1, and HRD model—to simulate the experiment and measure *V*_*m*_ decay in two-dimensional space. The FHN model is a simple excitable system model with two differential equations and a resting potential of *V*_*m*_ ≈ −1.5. We added the partial differential operators for the two-dimensional simulation to get the following equation set:

$$\begin{array}{c} \frac{\partial V_{m}}{\partial t}=V_{m}-\frac{V_{m}^{3}}{3}-W+J+D(\frac{\partial^{2}V_{m}}{\partial x^{2}}+\frac{\partial^{2}V_{m}}{\partial x^{2}}), \end{array}$$ (29)

$$\begin{array}{c} \frac{\partial W}{\partial t}=\epsilon \left(V_{m}+\beta -\gamma \times W\right). \end{array}$$ (30)

Here, the model has been put in dimensionless form, *W* represents the gating variable and *J* is the stimulus current, while *ϵ*, *β* and *γ* are constants. The values used for *ϵ*, *β* and *γ* were 0.03, 1.2 and 0.3, respectively.

**Fig 13:**
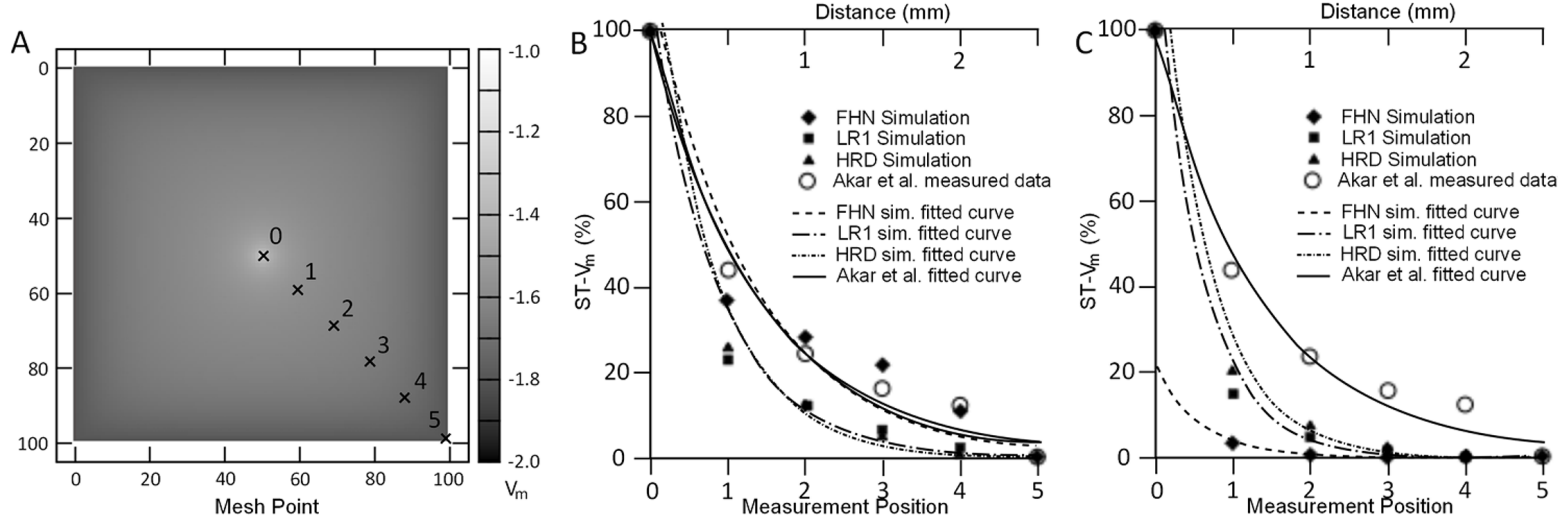
Results of the cell-to-cell coupling and membrane potential (*V*_*m*_) decay simulations using the FHN, LR1 and HRD model. A 100 × 100 mesh (A) was used for the FHN and LR1 simulations, while HRD simulations used a 50 × 50 mesh. A subthreshold stimulus was applied at the center of the mesh and the value of *V*_*m*_ was measured at equal intervals (cross marks) along the diagonal from the stimulation source (point 0) to the lower right corner (point 5). Two sets of simulations were done, each using a different boundary condition. The first set used a Dirichlet boundary condition where *V*_*m*_ was set at a fixed value along the boundary (B). The other set used Neumann boundary condition (C). Both sets of simulations were compared with the experimental data recorded by Akar et al. [34]. Akar et al. applied a subthreshold stimulus of 45 mV to a guinea pig’s heart and measurements were made for sites 0–2.5 mm from the stimulating electrode. The simulation and experimental data curves show the exponential decay of *V*_*m*_ as measurements move away from the site of stimulation.

The FTCS scheme was used to discretize the model equations in all the simulations, with time step Δ*t* = 0.001 ms and diffusion constant *D* = 200 mm^2^/ms. Simulation morphology was a 100 × 100 square mesh for the FHN and LR1 simulations (Fig 13). However, due to memory constraints in generating the program code, a smaller 50 × 50 mesh was used for the HRD simulations.

To simulate transduction, we put a fixed subthreshold stimulus in the center of the mesh. Values of *V*_*m*_ = −1.0 for the FHN simulations and and *V*_*m*_ = −75.0 millivolts (mV) for the LR1 and HRD simulations were used as stimulus. Two sets of experiments were done, one using Dirichlet boundary condition and another using Neumann boundary. For the simulations with Dirichlet boundary condition, arbitrary values of *V*_*m*_ = −1.7 for FHN and *V*_*m*_ = −87.0 mV for LR1 and HRD were assigned to the boundary nodes.

Simulation values of *V*_*m*_ was measured at even intervals along a diagonal from the center of the mesh to one of its corners (cross marks in Fig 13A). The membrane potential decay curve was plotted using the computed *V*_*m*_ ratios as described by Akar et al. Additional values were also gathered in between interval points to increase the accuracy of the fitted curve.

Fig 13A shows the distribution of *V*_*m*_ around the simulation source (point 0) and along the boundary for the FHN model simulation. Comparisons of simulation results with the measured data published by Akar et al. [34] are shown in Fig 13B for simulations with Dirichlet boundary conditions and Fig 13C for simulations with Neumann boundary conditions. The two sets of experiments using two different boundary conditions were done because the boundary condition used in the wet experiments was not clear to us. We used these results and superimposed the gathered experimental data and corresponding fitted curve of the published data to compare the decay curve of *V*_*m*_ in different biological function models and boundary conditions.

Results show that, with the exception of the FHN simulation with Dirichlet boundary conditions, the membrane potential decay in simulations were faster than in the published experiments. The decay curve of the more complicated models in LR1 and HRD were similar for both boundary conditions. FHN gives the best results probably because of its simplicity and it can be roughly fit to the data. LR1 and HRD on the other hand are are models of different species than guinea pig.

One thing we noticed while we were trying different values of *D* and Δ*t* was that the simulation results get closer to the experimental data as we increase *D*. Increasing the value of *D* does not lead to significant changes in simulation time but for every increase, we need to reduce Δ*t*. This is to guarantee stability for the diffusion operator and satisfy the condition Δ*t* ≤ Δ*x*/(2*dD*), where *d* is the system dimension (1, 2 or 3) and Δ*x* the space step [36]. The simulations shown in Fig 13 took up to five hours to complete. However, further reductions in the time step stretches the simulation time into days and even weeks. The simulations shown here were done to demonstrate our system’s ability to automatically handle boundary conditions and generate program code. The program code and raw data results for the coupling simulations mentioned in this section are included in the SourceForge page of our project [31].

### Software Utility

In this system, we focused on finite difference single-step replacement methods on regular mesh morphology. Although the system can generate program code for different types of boundary conditions and biological function models, it has limitations in the morphology size and number of equations it can handle. For the Luo-Rudy model simulation, there are eight differential and 31 arithmetic equations, with additional equations from all of the boundary instances in a 2D mesh. A simulation using a 100 × 100 mesh size would give more than 390,000 equations that would need to undergo dependency analysis. Dependency analysis for this number of equations would require a large allocated memory not available in most general-purpose computers.

In modern PDE simulation systems, modern methods such as finite element methods, operator splitting methods or mesh-free simulations are often used for their computational efficiency. Our system is nowhere near the capacity and speed of these modern methods, thus, our system is not useful in the research field, which requires top level PDE simulation performance. However, since our system generates easy to read and understand program codes without introducing human errors, the system will be useful for researchers who are not familiar with PDE simulation program codes or those who are not familiar with cell models. Researchers who may not be familiar with PDE simulations but are interested in evaluating the spatial distribution effect of certain phenomena can immediately start their simulation using simple models through our system. Since the program code is easy to understand, they can easily modify their model or governing equations. This may be helpful especially for those who are in the life science field and may encourage more researchers to utilize spatially-distributed models.

## Conclusions

In this study, we proposed a general method for applying finite difference schemes to partial differential equations on uniform rectilinear grids. Our system handles morphology and boundary condition information using markup language files. Through the proposed method, it is possible to automatically generate program code for cardiac electrophysiology simulations modelled by distributed parameter systems. Automatic handling of simultaneous equations and implicit finite difference methods is also implemented. This is done through the insertion of a Newton solver function into the generated code to solve for sets of simultaneous and nonlinear equations. Generation of the Newton solver code, however, takes an inordinate amount of time compared to the other parts of the code generation process and needs further optimization. This will be included in our future work as well as further improvements on our system’s design for handling bigger mesh sizes and faster code generation time.

## Supporting Information

S1 Input Samples TecML, CellML and RelML sample input files. (PDF)
