# Supplementary material for: Program Code Generator for Cardiac Electrophysiology Simulation with Automatic PDE Boundary Condition Handling
Source: PLoS One. 2015 Sep 10;10(9):e0136821. doi: 10.1371/journal.pone.0136821 (PMC4565589; doi:10.1371/journal.pone.0136821)
Supplement: S1 Input Samples — (PDF) [file pone.0136821.s001.pdf]

Sample Input Files

TecML File

```
<tecml>
<!--         FTCS_Diffusion_2D.tecml: forward-time center-space FDM scheme  -->
<!--        Single-step Replacement Scheme 2D array                         -->
<!--        Last edit:   20140108                                           -->
<!--        Variables: v = differential variables, y = arithmetic variables,-->
<!--                        z = constants, c = distributed parameters       -->

<variable name="v" type="recurvar" />
<variable name="y" type="arithvar" />
<variable name="z" type="constvar" />
<variable name="c" type="constvar" />

<variable name="t" type="timevar"/>
<variable name="x1" type="spatialvar"/>
<variable name="x2" type="spatialvar"/>

<variable name="deltat" type="constvar" />
<variable name="deltax1" type="constvar" />
<variable name="deltax2" type="constvar" />

<variable name="n" type="stepvar" />
<variable name="i" type="stepvar" />
<variable name="j" type="stepvar" />
<!--        <variable name="k" type="stepvar" />   -->

<math xmlns="http://www.w3.org/1998/Math/MathML">

        <apply><eq/>
                <ci> t </ci>
                <apply><selector/>
                        <ci> t </ci>
                        <ci> n </ci>
                </apply>
        </apply>

        <apply><eq/>
                <ci> v </ci>
                <apply><selector/>
                        <ci> v </ci>
                        <ci> n </ci>
                        <ci> i </ci>
                        <ci> j </ci>
```

```
        </apply>
</apply>

<apply><eq/>
        <ci> y </ci>
        <apply><selector/>
                <ci> y </ci>
                <ci> n </ci>
                <ci> i </ci>
                <ci> j </ci>
        </apply>
</apply>

<apply type="parameter-cond" parameter-id="0"><eq/>
        <ci> c </ci>
        <apply><selector/>
                <ci> c </ci>
                <ci> n </ci>
                <ci> i </ci>
                <ci> j </ci>
        </apply>
</apply>

<!-- Neumann boundary conditions at time n -->

<apply type="boundary-cond" boundary-id="1" location="left"><eq/>
        <apply><partialdiff/>
                <bvar><ci> x1 </ci></bvar>
                <ci> v </ci>
        </apply>
        <apply><minus/>
                <apply><selector/>
                        <ci> v </ci>
                        <ci> n </ci>
                        <ci> i </ci>
                        <ci> j </ci>
                </apply>
                <apply><selector/>
                        <ci> v </ci>
                        <ci> n </ci>
                        <apply><plus/>
                                <ci> i </ci>
                                <cn> 1 </cn>
                        </apply>
                        <ci> j </ci>
                </apply>
```

```
        </apply>
</apply>

<apply type="boundary-cond" boundary-id="2" location="right"><eq/>
        <apply><partialdiff/>
                <bvar><ci> x1 </ci></bvar>
                <ci> v </ci>
        </apply>
        <apply><minus/>
                <apply><selector/>
                        <ci> v </ci>
                        <ci> n </ci>
                        <ci> i </ci>
                        <ci> j </ci>
                </apply>
                <apply><selector/>
                        <ci> v </ci>
                        <ci> n </ci>
                        <apply><minus/>
                                <ci> i </ci>
                                <cn> 1 </cn>
                        </apply>
                        <ci> j </ci>
                </apply>
        </apply>
</apply>

<apply type="boundary-cond" boundary-id="4" location="top"><eq/>
        <apply><partialdiff/>
                <bvar><ci> x2 </ci></bvar>
                <ci> v </ci>
        </apply>
        <apply><minus/>
                <apply><selector/>
                        <ci> v </ci>
                        <ci> n </ci>
                        <ci> i </ci>
                        <ci> j </ci>
                </apply>
                <apply><selector/>
                        <ci> v </ci>
                        <ci> n </ci>
                        <ci> i </ci>
                        <apply><plus/>
                                <ci> j </ci>
                                <cn> 1 </cn>
```

```
                        </apply>
                </apply>
        </apply>
</apply>

<apply type="boundary-cond" boundary-id="8" location="bottom"><eq/>
        <apply><partialdiff/>
                <bvar><ci> x2 </ci></bvar>
                <ci> v </ci>
        </apply>
        <apply><minus/>
                <apply><selector/>
                        <ci> v </ci>
                        <ci> n </ci>
                        <ci> i </ci>
                        <ci> j </ci>
                </apply>
                <apply><selector/>
                        <ci> v </ci>
                        <ci> n </ci>
                        <ci> i </ci>
                        <apply><minus/>
                                <ci> j </ci>
                                <cn> 1 </cn>
                        </apply>
                </apply>
        </apply>
</apply>

<!-- The following boundary conditions are for Dirichlet condition -->

<apply type="boundary-cond" boundary-id="64"><eq/>
        <ci> v </ci>
        <apply><selector/>
                <ci> v </ci>
                <ci> n </ci>
                <ci> i </ci>
                <ci> j </ci>
        </apply>
</apply>

<apply><eq/>
        <apply><partialdiff/>
                <bvar><ci> x1 </ci><degree><cn> 2 </cn></degree></bvar>
                <degree><cn> 2 </cn></degree>
                <apply>
```

```
                <ci> v </ci>
            </apply>
        </apply>
        <apply><divide/>
            <apply><plus/>
                <apply><selector/>
                    <ci> v </ci>
                    <ci> n </ci>
                    <apply><plus/>
                        <ci> i </ci>
                        <cn> 1 </cn>
                    </apply>
                    <ci> j </ci>
                </apply>
                <apply><minus/>
                    <apply><times/>
                        <cn> 2 </cn>
                        <apply><selector/>
                            <ci> v </ci>
                            <ci> n </ci>
                            <ci> i </ci>
                            <ci> j </ci>
                        </apply>
                    </apply>
                </apply>
                <apply><selector/>
                    <ci> v </ci>
                    <ci> n </ci>
                    <apply><minus/>
                        <ci> i </ci>
                        <cn> 1 </cn>
                    </apply>
                    <ci> j </ci>
                </apply>
            </apply>
            <apply><times/>
                <ci> deltax1 </ci>
                <ci> deltax1 </ci>
            </apply>
        </apply>
    </apply>

    <apply><eq/>
        <apply><partialdiff/>
            <bvar><ci> x2 </ci><degree><cn> 2 </cn></degree></bvar>
            <degree><cn> 2 </cn></degree>
```

```
                <apply>
                        <ci> v </ci>
                </apply>
        </apply>
        <apply><divide/>
                <apply><plus/>
                        <apply><selector/>
                                <ci> v </ci>
                                <ci> n </ci>
                                <ci> i </ci>
                                <apply><plus/>
                                        <ci> j </ci>
                                        <cn> 1 </cn>
                                </apply>
                        </apply>
                        <apply><minus/>
                                <apply><times/>
                                        <cn> 2 </cn>
                                        <apply><selector/>
                                                <ci> v </ci>
                                                <ci> n </ci>
                                                <ci> i </ci>
                                                <ci> j </ci>
                                        </apply>
                                </apply>
                        </apply>
                        <apply><selector/>
                                <ci> v </ci>
                                <ci> n </ci>
                                <ci> i </ci>
                                <apply><minus/>
                                        <ci> j </ci>
                                        <cn> 1 </cn>
                                </apply>
                        </apply>
                </apply>
                <apply><times/>
                        <ci> deltax2 </ci>
                        <ci> deltax2 </ci>
                </apply>
        </apply>
</apply>

<apply><eq/>
        <apply><partialdiff/>
                <bvar><ci> t </ci></bvar>
```

```
                        <apply>
                                <ci> v </ci>
                        </apply>
                </apply>
                <apply><divide/>
                        <apply><minus/>
                                <apply><selector/>
                                        <ci> v </ci>
                                        <apply><plus/>
                                                <ci> n </ci>
                                                <cn> 1 </cn>
                                        </apply>
                                        <ci> i </ci>
                                        <ci> j </ci>
                                </apply>
                                <apply><selector/>
                                        <ci> v </ci>
                                        <ci> n </ci>
                                        <ci> i </ci>
                                        <ci> j </ci>
                                </apply>
                        </apply>
                        <ci> deltat </ci>
                </apply>
        </apply>

</math>

</tecml>
```

CellML File

```
<?xml version="1.0" encoding="utf-8"?>
<model name="fhn_diffusion_2D" cmeta:id="fhn" xmlns="http://www.cellml.org/cellml/1.0#"
  xmlns:cellml="http://www.cellml.org/cellml/1.0#"
  xmlns:cmeta="http://www.cellml.org/metadata/1.0#">
  <documentation xmlns="http://cellml.org/tmp-documentation">
  <article>
        <articleinfo>
           <title>The FitzHugh-Nagumo Simplified Cardiac Myocyte Model</title>
        </articleinfo>
  </article>
  </documentation>
  <units name="millisecond">
        <unit units="second" prefix="milli"/>
  </units>
```

```
<component name="Main">
<variable name="time" units="millisecond" initial_value="0"/>
<variable name="r" units="dimensionless" initial_value="0"/>
<variable name="v" units="dimensionless" initial_value="0"/>
<variable name="w" units="dimensionless" initial_value="0"/>
<variable name="zz" units="dimensionless" initial_value="0"/>
<variable name="beta" units="dimensionless" initial_value="1.2"/>
<variable name="gamma" units="dimensionless" initial_value="0.3"/>
<variable name="epsilon" units="dimensionless" initial_value="0.03"/>
<variable units="micrometer" name="x1" initial_value="1"/>
<variable units="micrometer" name="x2" initial_value="1"/>
<variable units="micrometer" name="D" initial_value="0.001"/>
<math xmlns="http://www.w3.org/1998/Math/MathML">
       <apply>
              <eq/>
              <ci>r</ci>
              <apply>
                 <times/>
                 <ci>v</ci>
                 <ci>v</ci>
                 <ci>v</ci>
              </apply>
       </apply>
       <apply>
              <eq/>
              <apply>
                 <diff/>
                 <bvar>
                        <ci>time</ci>
                 </bvar>
                 <ci>v</ci>
              </apply>
              <apply>
                 <plus/>
                 <apply>
                        <minus/>
                        <apply>
                               <minus/>
                               <ci>v</ci>
                               <apply>
                                      <divide/>
                                      <ci>r</ci>
                                      <cn cellml:units="dimensionless">3</cn>
                               </apply>
                        </apply>
                        <ci>w</ci>
```

```
          </apply>
          <ci>zz</ci>
<apply><times/>
<ci> D </ci>
<apply>
 <partialdiff/>
 <bvar><ci> x1 </ci><degree><cn> 2 </cn></degree></bvar>
 <degree><cn> 2 </cn></degree>
  <apply>
   <ci> v </ci>
  </apply>
</apply>
</apply>
<apply><times/>
 <ci> D </ci>
 <apply>
   <partialdiff/>
   <bvar><ci> x2 </ci><degree><cn> 2 </cn></degree></bvar>
   <degree><cn> 2 </cn></degree>
   <apply>
        <ci> v </ci>
   </apply>
 </apply>
</apply>
        </apply>
 </apply>
 <apply>
        <eq/>
        <apply>
           <diff/>
           <bvar>
                  <ci>time</ci>
           </bvar>
           <ci>w</ci>
        </apply>
        <apply>
           <times/>
           <ci>epsilon</ci>
           <apply>
                  <minus/>
                  <apply>
                         <plus/>
                         <ci>v</ci>
                         <ci>beta</ci>
                  </apply>
                  <apply>
```

```
                                <times/>
                                <ci>gamma</ci>
                                <ci>w</ci>
                         </apply>
                  </apply>
               </apply>
        </apply>
  </math>
  </component>
</model>
```

RelML File

```
<!--                 FHN_FTCS_Diffusion_2D.relml                           -->
<!-- Finite Difference solution in 2D for the FitzHugh-Nagumo model         -->
<!-- with diffusion PDE and Forward-Time Central Space Scheme (FTCS)        -->

<relml>

<tecml filename="model/TecMLPDE/FTCS_Diffusion_2D.tecml" />
<cellml filename="model/CellML/fhn_diffusion_2D.cellml"/>

<variable name="Main.time" tecmlname="t" init="0"/>
<variable name="Main.x1" tecmlname="x1" />
<variable name="Main.x2" tecmlname="x2" />

<variable name="deltat" tecmlname="deltat" init="0.01" />
<variable name="deltax1" tecmlname="deltax1" init="1" />
<variable name="deltax2" tecmlname="deltax2" init="1" />

<variable name="Main.v" tecmlname="v" init="-1.501250563778375"/>
<variable name="Main.w" tecmlname="v" init="-0.37621367749846896"/>
<variable name="Main.r" tecmlname="y" />
<variable name="Main.zz" tecmlname="z" />
<variable name="Main.epsilon" tecmlname="z" init="0.03" />
<variable name="Main.beta" tecmlname="z" init="1.2" />
<variable name="Main.gamma" tecmlname="z" init="0.3"/>
<variable name="Main.D" tecmlname="z" init="1"/>

<morphology name="2D LV wall model" filename="csv/morphology_square_100x100.csv" >
        <geometry geometry-id="1" tecmlname="solid" dimension="2">
                <mesh tecmlname="RECTILINEAR_GRID" sizex="100" sizey="100" sizez="1" spac
        </geometry>
</morphology>
```

```
<boundary-cond variablename="Main.v" filename="csv/boundary_square_100x100.csv" />
<!-- <parameter-cond variablename="Main.epsilon" filename="csv/dpEpsilon_square_100x100cs
<!-- <parameter-cond variablename="Main.zz" parameter-id="1" /> -->

<math>
        <apply type="boundary-cond" boundary-id="1" location="left"><eq/>
                <apply><partialdiff/>
                        <bvar><ci> Main.x1 </ci></bvar>
                        <ci> Main.v </ci>
                </apply>
                <cn> 0 </cn>
        </apply>

        <apply type="boundary-cond" boundary-id="2" location="right"><eq/>
                <apply><partialdiff/>
                        <bvar><ci> Main.x1 </ci></bvar>
                        <ci> Main.v </ci>
                </apply>
                <cn> 0 </cn>
        </apply>

        <apply type="boundary-cond" boundary-id="4" location="top"><eq/>
                <apply><partialdiff/>
                        <bvar><ci> Main.x2 </ci></bvar>
                        <ci> Main.v </ci>
                </apply>
                <cn> 0 </cn>
        </apply>

        <apply type="boundary-cond" boundary-id="8" location="bottom"><eq/>
                <apply><partialdiff/>
                        <bvar><ci> Main.x2 </ci></bvar>
                        <ci> Main.v </ci>
                </apply>
                <cn> 0 </cn>
        </apply>
</math>

</relml>
```
